# Comprehensive Analysis Revealed the Potential Roles of N^6^-Methyladenosine (m^6^A) Mediating *E. coli* F18 Susceptibility in IPEC-J2 Cells

**DOI:** 10.3390/ijms232113602

**Published:** 2022-11-06

**Authors:** Zhengchang Wu, Yifu Wang, Tong Li, Li Yang, Jian Jin, Shenglong Wu, Wenbin Bao

**Affiliations:** 1 Key Laboratory for Animal Genetics, Breeding, Reproduction and Molecular Design of Jiangsu Province, College of Animal Science and Technology, Yangzhou University, Yangzhou 225009, China; 2 Joint International Research Laboratory of Agriculture & Agri-Product Safety, Yangzhou University, Yangzhou 225009, China

**Keywords:** N^6^-methyladenosine (m^6^A), IPEC-J2, *E. coli* F18, susceptibility

## Abstract

Post-weaning diarrhea caused by enterotoxigenic *Escherichia coli* F18 *(E. coli* F18) causes significant economic losses for pig producers. N^6^-methyladenosine (m^6^A) is a highly abundant epitranscriptomic marker that has been found to be involved in regulating the resistance of host cells to pathogenic infection, but its potential role in *E. coli* F18-exposed intestinal porcine epithelial cells (IPEC-J2) remains undetermined. Here, we demonstrated that m^6^A and its regulators modulate *E. coli* F18 susceptibility. Briefly, we revealed that the Wilms’ tumor 1-associating protein (*WTAP*) expressions were markedly elevated in IPEC-J2 cells upon *E. coli* F18 exposure. *WTAP* are required for the regulation of *E. coli* F18 adhesion in IPEC-J2 cells. Additionally, *WTAP* knockdown significantly suppressed m^6^A level at N-acetyllactosaminide beta-1,6-N-acetylglucosaminyl-transferase (*GCNT2*) 3′UTR, resulting in the enhancement of TH N^6^-methyladenosine RNA binding protein 2 (YTHDF2)-mediated *GCNT2* mRNA stability. Subsequently, the altered *GCNT2* expressions could inhibit the glycosphingolipid biosynthesis, thus improving resistance to *E. coli* F18 infection in IPEC-J2. Collectively, our analyses highlighted the mechanism behind the m^6^A-mediated management of *E. coli* F18 susceptibility, which will aid in the development of novel approaches that protect against bacterial diarrhea in piglets.

## 1. Introduction

Piglets’ bacterial diarrhea is one of the most common intestinal inflammatory diseases, which becomes the biggest contributor to financial loss to pig farmers everywhere. Enterotoxigenic *Escherichia coli* F18 (ETEC F18) is a major pathogen that induces post-weaning piglet diarrhea, and it strongly interacts with the intestinal porcine epithelial cell (IPEC-J2) using pili [1]. F18 fimbriae are commonly found in *E. coli* extracted from weaned piglets, and they can be categorized into two major antigenic variants, F18ab and F18ac. F18ab is closely related to the Stx2e-producing strains that cause edema disease (ED). However, many F18ab isolates (10 of 11 isolates, 90.9%) are also found in ETEC. F18ac is associated with enterotoxigenic bacteria that encodes STa and STb, which causes post-weaning diarrhea (PWD) [2]. It is well reported that the *E. coli* F18 pathogenicity is based on the levels of corresponding receptors that express on the brush border of the piglet IPEC-J2, resulting in the production of LPS-enterotoxin and causing diarrhea [3]. Thus, resistance against the *E. coli* F18 invasion depends on the intestinal immunity and F18-receptor content in pigs. Recently, several studies analyzed the association between immune-related genes (*CD14, TLR5*) [4,5], or fucosetransferase genes (*FUT2, FUT3*) [6,7] and *E. coli* F18 susceptibility in piglets. Nevertheless, the mechanism behind piglets’ resistance to *E. coli* F18 invasion is not fully understood. Therefore, it is necessary to carry out a comprehensive and in-depth investigation into this process.

Emerging evidence revealed that chemical-based RNA modifications modulate gene expression. The N^6^-methyladenosine (m^6^A) methylation is relatively common among mammalian mRNAs, and it is strongly conserved in eukaryotes from yeast to humans [8,9]. Several reports demonstrated that the m^6^A transcript or non-coding RNA modification can regulate RNA fate and activity. Generally, m^6^A is deposited on RNAs via the m^6^A methyltransferase complex (METTL3/METTL14/WTAP), reversed using demethylases (FTO/ALKBH5), and identified by the m^6^A interacting proteins (YTHDF1-3/YTHDC1,2) [10]. Molecular studies revealed that this epigenetic alteration modulates RNA stability, translation, alternative splicing, and nuclear export [11,12,13,14]. Thus, m^6^A are intricately related to numerous physiological processes, namely, tissue development, tumorigenesis and cell division [15]. Although numerous investigations have examined the association between m^6^A modifications and physical traits in humans, great progress has been made in the study of m^6^A regulating pig characters, including fat deposition [16], lipid metabolism [17], stem cell differentiation [18], liver development [19], etc. Nonetheless, litter studies have not yet characterized the association between m^6^A modifications and piglets’ bacterial diarrhea, underscoring a novel direction for the ongoing epigenetic studies on economically important livestock.

In general, the intestinal porcine epithelial cell (IPEC-J2) is an efficient cell model for the examination of the underlying mechanism behind the host anti-*E. coli* F18 pathogen [7,20]. In addition, multiple studies have revealed that the IPEC-J2 exposure to *E. coli* triggers oxidative damage and inflammatory responses, which results in enhancing apoptotic cell death and diminishing functionality [21,22,23]. Nevertheless, the explicit biological role of m^6^A regulators in conferring resistance against *E. coli* F18-based infection in IPEC-J2 cells remains undetermined. Herein, we demonstrated a crucial role of m^6^A and its regulators in affecting the susceptibility of IPEC-J2 cells to *E. coli* F18 exposure. We revealed that m^6^A regulator (WTAP) depletion significantly enhanced the adhesive capacity of *E. coli* F18-fimbriae to IPEC-J2 cells. Moreover, we showed that WTAP regulated target expression by modulating mRNA stability using an m^6^A-YTHDF2-based system. In short, we highlighted the mechanism behind the m^6^A-mediated regulation of resistance to *E. coli* F18-based infection in IPEC-J2 cells and will provide valuable targets that induce resistance to bacterial diarrhea in pigs.

## 2. Results

### 2.1. Upregulated WTAP Expressions in IPEC-J2 Cells after E. coli F18 Exposure

**Figure 1:**
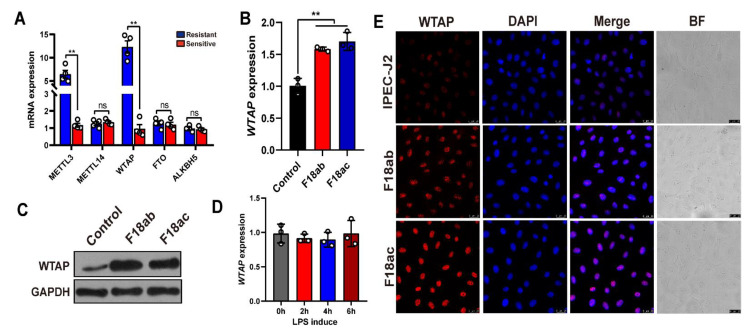
Expression analysis of m^6^A methyl markers in IPEC-J2 cells upon *E. coli* F18 exposure. (**A**) qPCR validation of *METTL3, WTAP, METTL14, FTO, ALKBH5* expression in duodenum tissues between F18-resistant (*n* = 4) and -sensitive piglets (*n* = 4). (**B**,**C**) Expression changes of WTAP in F18ab/ac-stimulated IPEC-J2 cells by using qPCR and Western blotting analysis. (**D**) Expression changes of *WTAP* in LPS-induced IPEC-J2 cells. IPEC-J2 cells were treated with 0.1 μg/mL LPS for 0, 2, 4, and 6 h. (**E**) Immunofluorescence analysis of WTAP in IPEC-J2 upon *E. coli* F18ab/ac exposure. Scale bar, 25 μm. All data are presented as the mean ± SD, ns *p* > 0.05, ** *p* < 0.01.

To preliminarily examine the role of m^6^A methyl markers in resistance to *E. coli* F18 infection in piglets, we detected the levels of three m^6^A writers (METTL3, METTL14, and WTAP) and two m^6^A erasers (ALKBH5 and FTO) in the duodenum of *E. coli* F18-sensitive and -resistant piglets. Based on our qPCR analysis (Figure 1A), the *METTL3* and *WTAP* expressions were markedly elevated in the resistant versus sensitive piglets (*p* < 0.01). We next assessed the levels of *WTAP* expressions in IPEC-J2 cells following *E. coli* F18 exposure. We revealed that both *WTAP* transcript and protein levels were considerably high in the F18ab/ac-treated cells (Figure 1B,C, *p* < 0.01). The lipopolysaccharide (LPS) layer in *E. coli* stabilizes the outer membrane of the bacteria [24]. Studies revealed that the IPEC-J2-exposed LPS triggers cell immune responses [5,25]. Our findings demonstrated that the *WTAP* expression showed no significant change in IPEC-J2 cells following a LPS induction (Figure 1D, *p* > 0.05). These results indicated that *WTAP* did not have a potential role in modulating *E. coli* F18-induced immune response but may be involved in other aspects of regulation. Moreover, immunofluorescence analysis revealed that the F18ab/ac-bacteria stimulation enhanced WTAP levels in the IPEC-J2 cells (Figure 1E). 

### 2.2. WTAP Is Required for the E. coli F18-Based Adhesion Regulation in IPEC-J2 Cells

**Figure 2:**
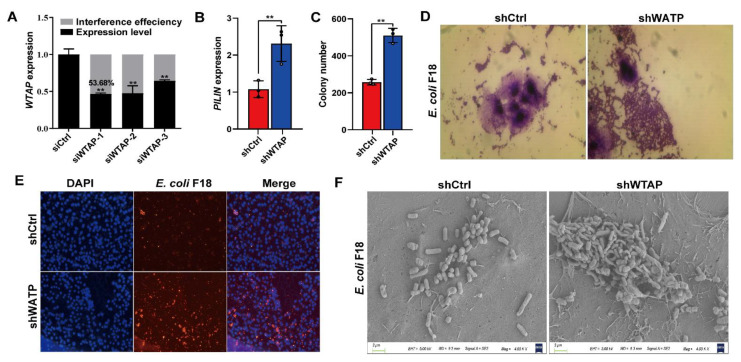
*WTAP* is required for affecting *E. coli* F18 adhesion in IPEC-J2 cells. (**A**) Knockdown efficiency of *WTAP* gene in IPEC-J2 cells was determined using qRT-PCR. (**B**) Expression detection of *E. coi* F18 fimbriae gene (*PILIN*) via relative quantification in *WTAP*-silenced IPEC-J2 cells. (**C**) Colony number of *E. coi* F18 fimbria adhering to IPEC-J2 cells were evaluated in treatment group. (**D**) Gram staining assay, an optical microscope (400×, scale bar = 100 μm) was used to observe the IPEC-J2 cells. (**E**) Indirect immunofluorescence assay, blue fluorescence indicates nuclear staining via DAPI; red fluorescence indicates staining with the anti-*E. coli* antibody. Cells were observed under a fluorescence microscope (100×, scale bar = 1 mm). (**F**) Scanning electron microscopy (SEM) assay, cells were observed under a scanning electron microscope (2000×). All data are presented as the mean ± SD, ** *p* < 0.01.

To elucidate the mechanism behind the *WTAP*-mediated regulation of *E. coli* F18 susceptibility, we assessed how *WTAP* levels modulated the adhesive capacity of F18ac-expressing fimbriae to IPEC-J2 cells. First, we generated an overexpression vector and designed three siRNA vectors for the *WTAP* gene. The optimal interference efficiency was 53.68% in IPEC-J2 cells with si*WTAP* vectors (Figure 2A) as evidenced by qPCR verification. F18-fimbriae protein (*PILIN*) expression detection (Figure 2B) and colony counting (Figure 2C) revealed a significantly higher quantity of bacteria adhering to the IPEC-J2 cells in the si*WTAP* group (*p* < 0.01). In addition, we also assessed the adhesive capacity of *E. coli* F18 to IPEC-J2 cells using gram staining (Figure 2D), indirect immunofluorescence (Figure 2E) and scanning electron microscope (Figure 2F). Consistently, the results revealed that the *WTAP* knockdown markedly increased the adhesion level of *E. coli* F18 to IPEC-J2 cells. Thus, our findings highlighted the significance of *WTAP* in regulating *E. coli* F18 invasion and confirmed that *WTAP* deficiency markedly enhanced *E. coli* F18 adhesion to IPEC-J2 cells.

### 2.3. GCNT2 3′UTR Mediates WTAP-m^6^A Regulation

**Figure 3:**
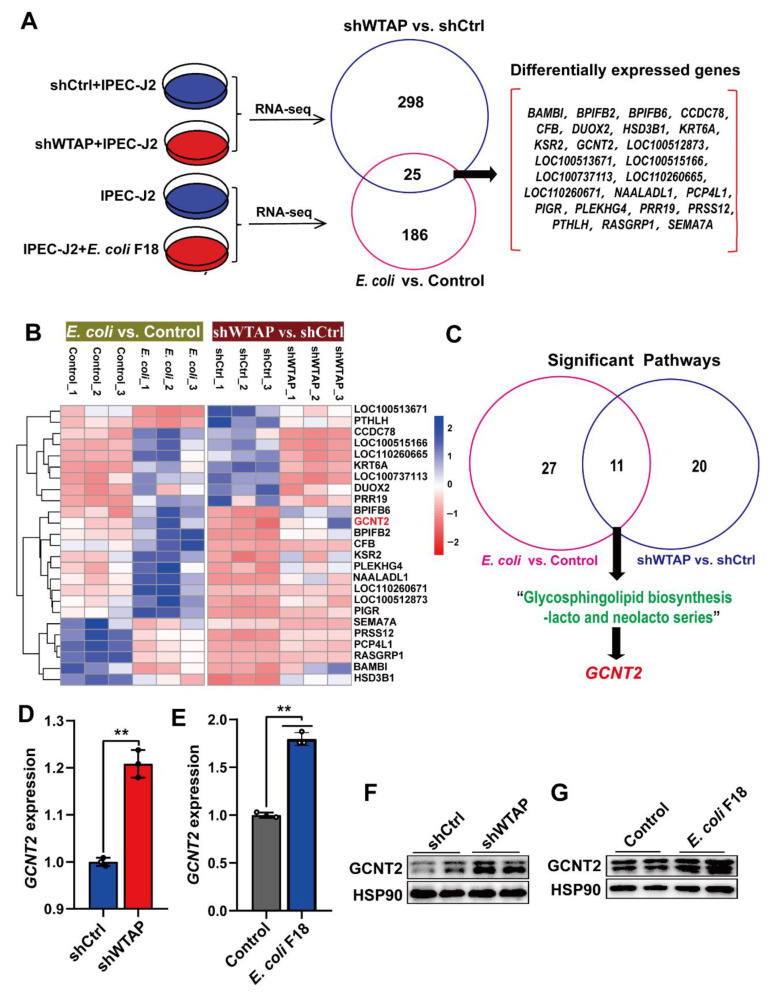
Transcriptome sequencing identified *GCNT2* as a WTAP downstream target. (**A**) Venny analysis of differentially expressed genes (DEGs) in IPEC-J2 from shWTAP and shCtrl group, *E. coli* and Control group by comparative transcriptome sequencing. (**B**) Hierarchical clustering analysis of common DEGs between shWTAP vs. shCtrl and *E. coli* vs. Control. (**C**) Venny analysis of significant pathways that DEGs were involved in. (**D**) qPCR analysis of GCNT2 expression in IPEC-J2 cells transfected with control and shWTAP vector. (**E**) qPCR analysis of the expression change of GCNT2 in IPEC-J2 cells after *E. coli* F18 infection. (**F**,**G**) Western blotting analysis of GACN2 expression in IPEC-J2 cells after WTAP knockdown or *E. coli* F18 infection. All data are presented as the mean ± SD, ** *p* < 0.01.

To understand the underlying mechanism by which *WTAP* regulates *E. coli* F18 susceptibility in IPEC-J2 cells, we employed a comparative transcriptome sequencing to highlight the transcriptional differences following *WTAP* knockdown or *E. coli* F18 infection. Overall, we identified 323 differentially expressed genes (DEGs) between the shWTAP and shCtrl groups (Figure S1), and 211 DEGs between the *E. coli* F18-treated and control samples (Figure S2). Interestingly, 25 common DEGs were screened in both the shWTAP vs. shCtrl and *E. coli* vs. control groups (Figure 3A,B). To further assess the potential biological impact of DEGs in *WTAP* knockdown or *E. coli*-treated cells, KEGG pathway enrichment analyses were performed. Our analysis revealed that the DEGs transcripts from the shWTAP vs. shCtrl and *E. coli* vs. control groups were involved in 11 common signaling pathways (Table S3), including a glycosphingolipid biosynthesis-lacto and neolacto series (Figure 3C), which is likely linked to the *E. coli* F18 receptor formation [26,27]. Among the 25 common DEGs, *GCNT2* serves as a critical molecule participating in the glycosphingolipid biosynthesis pathway. Based on our qRT-PCR data (Figure 3D,E), the *GCNT2* levels were markedly upregulated in both *WTAP* knockdown and *E. coli* F18-treated cells (*p* < 0.01). Similarly, our Western blot results revealed (Figure 3F,G) that the *GCNT2* protein levels were also significantly enhanced in these treated cells. Thus, our findings preliminarily identified *GCNT2* as a downstream target of *WTAP* by using transcriptome sequencing.

**Figure 4:**
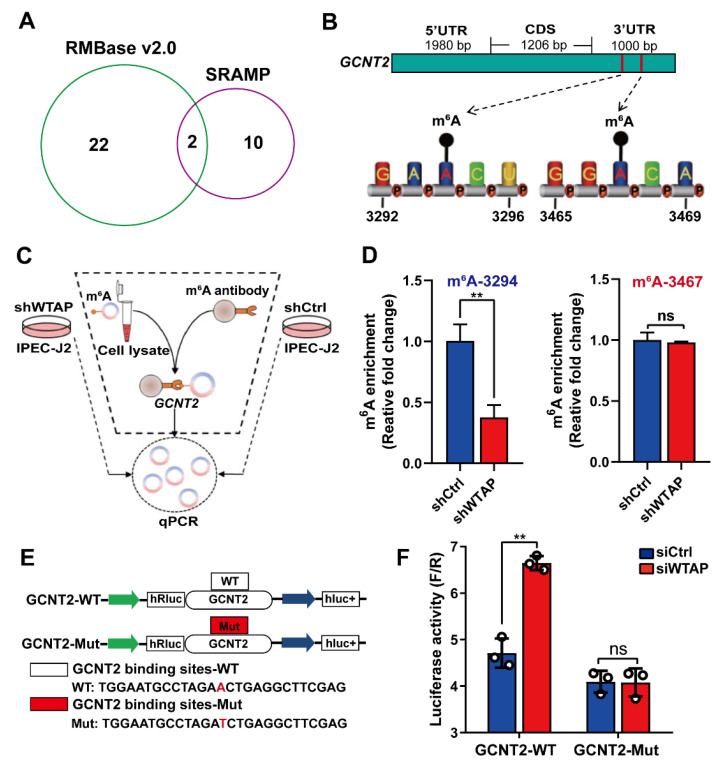
WTAP regulates *GCNT2* expression via m^6^A methylation. (**A**) Predicted m^6^A sites in *GCNT2* from overlapping results of SRAMP and RMBase v2.0 software. (**B**) Schematic diagram of predicted m^6^A sites in the 3′UTR of *GCNT2* gene. (**C**) Flow diagram of m^6^A-specific immunoprecipitation (MeRIP) assays. (**D**) MeRIP assays for m^6^A-modified *GCNT2* in shWTAP and shCtrl cell lines. (**E**) Construction of luciferase vector with *GCNT2* wild-type 3′UTR (WT) or mutant-type 3′UTR (Mut). (**F**) Relative activity of the wild-type or mutant *GCNT2* 3′UTR firefly luciferase reporter in IPEC-J2 cells transfected with control or WTAP siRNAs. All data are presented as the mean ± SD, ** *p* < 0.01, ns *p* > 0.05.

To further explore whether m^6^A modification are essential for the WTAP-based regulation of *GCNT2* expression, we employed the SRAMP [28] and RMBase v2.0 [29] to predict m^6^A sites. We identified two m^6^A sites (3294, 3467) in the 3′UTR of the *GCNT2* gene (Figure 4A,B). Importantly, using the m^6^A-specific immunoprecipitation assay (Figure 4C), we revealed a significant downregulation of m^6^A levels in the 3′UTR region (3294) of *GCNT2* in the *WTAP* knockdown IPEC-J2 cells, relative to mock-vehicle controls (*p* < 0.01). In contrast, the m^6^A level at the 3′UTR site (3467) displayed no significant change in the WTAP-silenced cells (Figure 4D, *p* > 0.05). We also conducted GCNT2-DLR and mutagenesis assays in the WTAP knockdown cells (Figure 4E). Based on our results, *WTAP* knockdown markedly enhanced bioluminescence of reporter carrying the *GCNT2* 3′UTR-WT fragment, compared to controls (Figure 4F). Taken together, our results confirmed that the *GCNT2* transcript was a functionally important substrate of WTAP.

### 2.4. WTAP Reduces GCNT2 mRNA Stability through an m^6^A-YTHDF2-Regulated Manner

**Figure 5:**
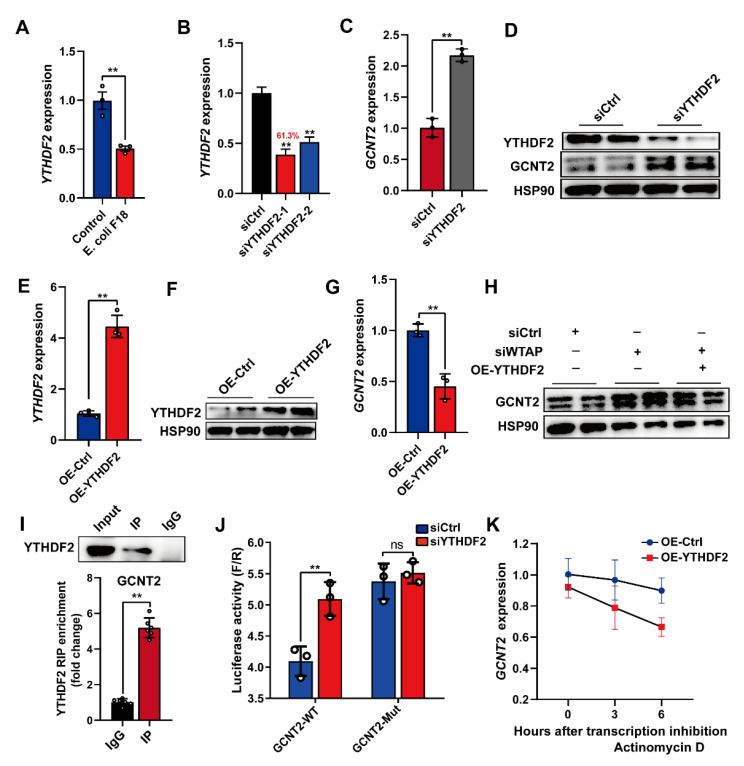
Silencing of WTAP enhanced *GCNT2* mRNA stability via YTHDF2-dependent mechanism. (**A**) qPCR detection of YTHDF2 expression in IPEC-J2 cells upon *E. coli* F18 exposure. (**B**) Knockdown efficiency of YTHDF2 gene in IPEC-J2 was assessed using qPCR. (**C**,**D**) qPCR and Western blot analysis of GCNT2 expression in control and YTHDF2-knockdown IPEC-J2 cells. (**E**,**F**) Overexpression efficiency of YTHDF2 gene in IPEC-J2 cells was determined using qPCR and Western blotting. (**G**) qPCR analysis of GCNT2 expression in control and YTHDF2-overexpressed IPEC-J2 cells. (**H**) Western blot analysis of GCNT2 expression in control, WTAP-knockdown, siWTAP + YTHDF2-overexpressed IPEC-J2 cells. (**I**) RIP assays of YTHDF2 association with IKBKG. Nuclear lysates of IPEC-J2 cells were immunoprecipitated with control mouse IgG or anti-histone YTHDF2 antibody, and the enrichment of GCNT2 was analyzed by using qPCR. (**J**) Relative luciferase activity of wild-type (WT) or mutant-type (Mut) GCNT2-3′UTR luciferase reporter in IPEC-J2 transfected with control or YTHDF2 siRNAs. (**K**) mRNA stability analysis of GCNT2 mRNA in control, YTHDF2-overexpressed IPEC-J2 treated with actinomycin D for 3 and 6 h. All data are presented as the mean ± SD, ** *p* < 0.01, ns *p* > 0.05.

Furthermore, we examined the m^6^A-mediated regulation of *GCNT2*. Based on published reports, the m^6^A “reader” protein-like YTHDF2 employs m^6^A modification to mediate degradation of m^6^A-rich transcripts [11]. This suggests that the YTHDF2 protein may recognize and interact with the m^6^A-rich *GCNT2* transcript. To examine this hypothesis, we detected the mRNA levels of YTHDF2 in *E. coli* F18-treated IPEC-J2 cells, and its expression demonstrated significant downregulation in IPEC-J2 cells upon *E. coli* F18 exposure (Figure 5A, *p* < 0.01). Subsequently, we established YTHDF2-silenced IPEC-J2 cells with a knockdown efficiency of 61.3% (Figure 5B). Upon *YTHDF2* knockdown, the *GCNT2* mRNA and protein expressions were markedly elevated in IPEC-J2 cells (Figure 5C,D, *p* < 0.01). We next assessed the overexpression efficiency of YTHDF2 in IPEC-J2 cells incorporated with the pcDNA3.1-YTHDF2, using qPCR and Western blot analyses (Figure 5E,F). YTHDF2 overexpression markedly reduced *GCNT2* transcript levels in IPEC-J2 cells (Figure 5G). Interestingly, the *WTAP* knockdown-mediated regulation of GCNT2 protein expression was reversed in cells with *YTHDF2* overexpression (Figure 5H), thus indicating that YTHDF2 modulates *GCNT2* expression. Likewise, RIP-qPCR analysis confirmed that *GCNT2* acts as a target of YTHDF2 (Figure 5I). Furthermore, *YTHDF2* overexpression markedly enhanced bioluminescence of luciferase reporters carrying the GCNT2 3′UTR-WT fragment (Figure 5J, *p* < 0.01). To further elucidate the importance of mRNA stability in the YTHDF2-mediated regulation of *GCNT2* expression, we evaluated expression alterations of *GCNT2* mRNA in the transcriptional inhibitor (actinomycin D) treated IPEC-J2 cells with carrying the control or *YTHDF2* overexpression vector. We clearly demonstrated that YTHDF2 overexpression obviously diminished the half-life of GCNT2 mRNAs (Figure 5K). Given these data, it was obvious that YTHDF2 was critical in modulating the WTAP-mediated action on *GCNT2* expression by affecting mRNA stability.

### 2.5. WTAP Potentially Regulates E. coli F18 Receptor Formation by Inhibiting the GCNT2-Mediated Glycosphingolipid Biosynthesis

**Figure 6:**
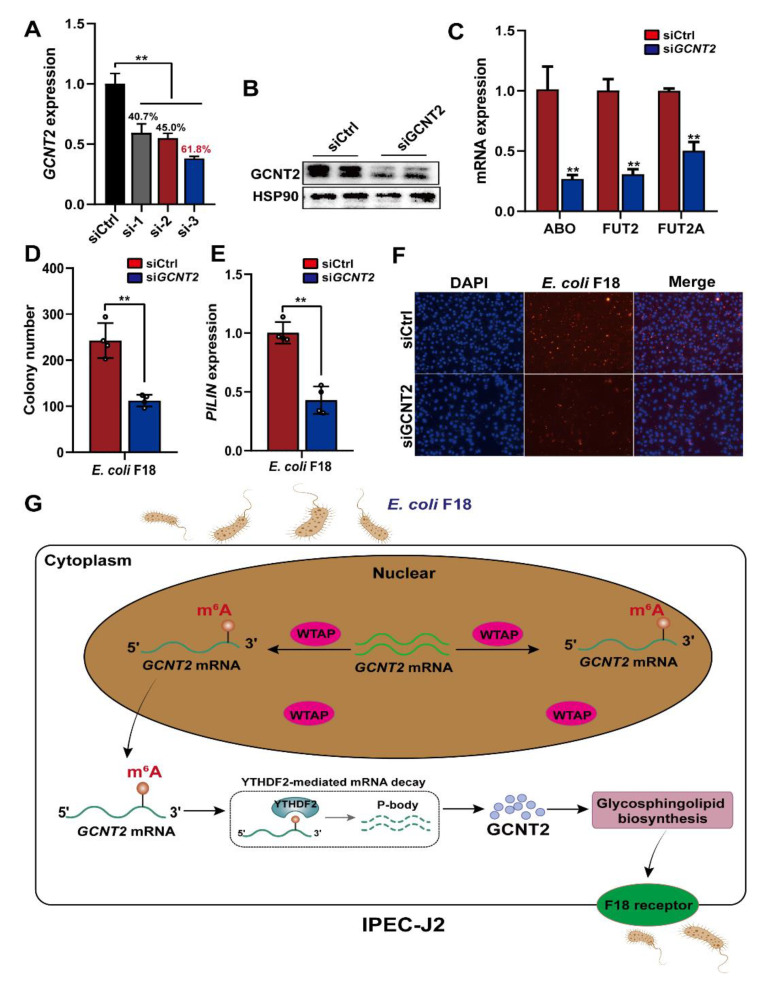
WTAP affects the formation of *E. coli* F18 receptor by inhibiting glycosphingolipid biosynthesis pathway. (**A**,**B**) Knockdown efficiency of *IKBKG* gene in IPEC-J2 was assessed using qPCR and Western blot analysis. (**C**) Expression detection of glycosphingolipid biosynthesis-related genes in IPEC-J2 cells with or without GCNT2 knockdown. (**D**–**F**) Effect of *GCNT2* knockdown on the ability of *E. coi* F18 fimbria to adhere to IPEC-J2 cells was analyzed by colony counting, *PILIN* expression detection, indirect immunofluorescence assay. (**G**) A working model summarizing the mechanism of m^6^A modification and its modulators in regulating *E. coli* F18 susceptibility in IPEC-J2 cells. m^6^A methyltransferase WTAP increases the m^6^A levels of GCNT2 mRNA, leading to WTAP attenuating the YTHDF2-dependent mRNA stability of GCNT2, WTAP thereby restraining the formation of *E. coli* F18 receptor by inhibiting GCNT2-mediated glycosphingolipid biosynthesis, resulting in the resistance of IPEC-J2 to *E. coli* F18 infection. All data are presented as the mean ± SD, ** *p* < 0.01.

To further elucidate the GCNT2-mediated regulation of *E. coli* F18 susceptibility in IPEC-J2 cells, we confirmed that *GCNT2* was a target of WTAP and it participated in the glycosphingolipid biosynthesis pathway (Figure 3C). Studies revealed that the glycosphingolipid biosynthesis was closely associated with F18 receptor formation [26,27]. Thus, we speculated that WTAP potentially regulates *E. coli* F18 receptor formation via the GCNT2-mediated glycosphingolipid biosynthesis. Thus, we constructed three siRNA vectors of *GCNT2* in IPEC-J2 cells, with an optimal knockdown efficiency of 61.8% (Figure 6A). Our Western blot analysis revealed that the GCNT2 protein expression was obviously decreased in the IPEC-J2 cells after *GCNT2* knockdown (Figure 6B). Moreover, *GCNT2* knockdown significantly reduced the expression of glycosphingolipid biosynthesis-related genes, including fucosyltransferase 2 (*FUT2*), galactoside 2-alpha-L-fucosyltransferase 2 (*FUT2A*), alpha 1-3-N-acetylgalactosaminyltransferase, and alpha 1-3-galactosyltransferase (*ABO*) (Figure 6C). Moreover, *GCNT2* knockdown considerably diminished the adhesive capacity of *E. coli* F18 to IPEC-J2 cells, as assessed by colony counting, *PILIN* expression, and indirect immunofluorescence assay (Figure 6D–F). Taken together, our findings indicated that *GCNT2* served a crucial function in controlling *E. coli* F18 adhesion via the regulation of glycosphingolipid biosynthesis.

## 3. Discussion

To successfully utilize IPEC-J2 cells in simulating the phenomenon of *E. coli* F18 adhesion in vitro, it is crucial to enhance our comprehension of the underlying mechanisms behind the m^6^A-mediated regulation of IPEC-J2 cells following *E. coli* F18 exposure. Herein, we identified an important mechanism whereby m^6^A modification regulates *E. coli* F18 susceptibility by modulating the NF-κB and glycosphingolipid biosynthesis signaling via the WTAP-m^6^A-based and YTHDF2-dependent post-transcriptional regulations (Figure 6G). Briefly, WTAP enhances the m^6^A status in *GCNT2* transcripts, which attenuates the WTAP-induced YTHDF2-dependent mRNA stability of *GCNT2*, thereby restraining the WTAP-based synthesis of *E. coli* F18 receptor by inhibiting the GCNT2-mediated glycosphingolipid biosynthesis. Collectively, these activities enhance resistance to *E. coli* F18 infection.

Functionally, m^6^A modification control numerous biological processes, such as metabolism, differentiation, and disease pathology [30]. Prior investigations demonstrated m^6^A as a critical contributor to host resistance of multiple pathogen/microbial infections [31,32,33,34]. METTL3 is a methyltransferase that accelerates m^6^A synthesis, which regulates viral replication and *E. coli* infection. Hao et al. (2019) reported that the host m^6^A complex METTL3 binds to viral proteins, and controls EV71 replication [35]. Zong et al. (2021) reported that the enterotoxigenic *E. coli* infection induces enteric defensin expression via the FOXO6-METTL3-m^6^A-GPR161 signaling axis [36]. WTAP is critical for m^6^A deposition and aids in the METTL3-METTL14 heterodimer heterodimer localizing to transcription sites [37,38]. Recent studies documented that the WTAP-mediated m^6^A modification critically regulates viral infection [39,40]. However, the potential contribution of WTAP regulating bacterial infection and its underlying mechanisms remain largely unknown. Our findings demonstrated that WTAP depletion enhanced the adhesive capacity of *E. coli* F18 fimbria to IPEC-J2 cells. In this study, we found that WTAP serves critical roles in the formation of E. coli F18 receptor via regulating GCNT2-based glycosphingolipid biosynthesis. In IPEC-J2 cells upon E. coli exposure, the *E. coli* F18 adhesion caused the upregulated expression of *WTAP* gene and activated the synthesis of F18 receptor. Given this evidence, we identified that the m^6^A methyl marker (WTAP) strongly modulated resistance to *E. coli* F18 infection.

It is well reported that m^6^A modification is primarily enforced by particular m^6^A-interacting proteins called m^6^A readers [15]. Given this scenario, some signaling pathways may be simultaneously modulated by m^6^A and its multiple m^6^A readers. Wu et al. (2019) demonstrated that m^6^A modulates the pluripotency of porcine pluripotent stem cells by influencing the SOCS3/JAK2/STAT3 axis in a YTHDF1/YTHDF2-regulated fashion [18]. Yao et al. (2019) reported that METTL3 suppresses BMSC adipogenic differentiation via modulation of the JAK1/STAT5/C/EBPβ axis in an m^6^A-YTHDF2-regulated fashion [41]. YTHDF2 recognizes and destabilizes m^6^A-rich transcripts [11], but YTHDF1 and YTHDF3 modulate transcript translation and protein synthesis [12,42]. Because m^6^A and the YTH domain family are ubiquitous among eukaryotes and they strongly regulate multiple biological processes, we proposed that the m^6^A interacting proteins serve discrete functions in the m^6^A-mediated regulation of *E. coli* susceptibility. In this study, we demonstrated that WTAP attenuated *GCNT2* mRNA stability and inhibited glycosphingolipid biosynthesis in a m^6^A-YTHDF2-dependent manner. Given this evidence, our research revealed crucial roles of YTHDF2 in *E. coli* F18 susceptibility-related gene regulation in IPEC-J2 cells. Du et al. (2016) reported that m^6^A reader protein YTHDF2 recruits the CCR4-NOT deadenylase complex by directly interacting with the superfamily homology (SH) domain of CNOT1, the scaffolding subunit of the complex, to initiate the deadenylation and decay of m6A-containing mRNAs [43]. In this study, the mechanism of YTHDF2 interacting with GCNT2 should still be further explored. WTAP is a Wilms’ tumor 1 (WT1) related protein that is necessary for numerous physiological processes, such as G2/M transition and pre-mRNA splicing [44,45,46]. Moreover, multiple reports highlighted the essential role of WTAP in the progression of cancers [47]. However, the m^6^A-based physiological function in IPEC-J2 is not yet determined. Here, we reported that WTAP affected the YTHDF2-based mRNA stability of GCNT2 in E. coli F18-exposed IPEC-J2 cells. It was reported that the mRNA stability of cyclin A2 and CDK2 regulated by WTAP contributed to the variation of mitotic cycle transition [46,48]. Taken together, our findings for the first time revealed the WTAP-involved m^6^A mechanism in IPEC-J2 cells following *E. coli* F18 exposure. 

## 4. Materials and Methods 

### 4.1. Reagents

The following antibodies were employed in immunoprecipitation (IP), indirect immunofluorescence assay (IFA) and Western blot assays: YTHDF2 (ab220163, rabbit, 1:30), WTAP (ab195380, rabbit, 1:1000), *E. coli* (ab137967, rabbit, 1:200) and GAPDH (ab9485, rabbit, 1:2500), HSP90 (ab59459, mouse, 1:500) were acquired from abcam (Shanghai, China). GCNT2 (Q06430, rabbit, 1:1000, Abcepta Biotech Ltd. Co., Suzhou, China); m^6^A antibody (56593, rabbit, 1:1000 Cell Signaling Technology, Boston, MA, USA); IgG (Q6005, rabbit, 1:1000 Dia-An Biotech, Wuhan, China).

### 4.2. Ethics Statement

All experimental protocols received ethical approval from the Yangzhou University (Pig: SYXK (Su) 2012-0029) and abided by the ethical standards of the People’s Republic of China. Our research did not warrant any other permission or approval.

### 4.3. Experimental Animals

Eight Meishan piglets (Kunshan Conservation Ltd., Suzhou, China) were used for these experiments. In prior studies, we obtained four *E. coli* F18-resistant and four *E. coli* F18-sensitive piglets by challenging with the *E. coli* F18 strain, and identifying via assays, such as bacterial counting, histopathological, and in vitro adherence assays of IPEC-J2 cells [5]. All piglet sacrifices were performed humanely via an intravenous administration of pentobarbital sodium, prior to the collection of duodenal tissues and storage in liquid nitrogen for additional use.

### 4.4. Cell Culture and E. coli F18 Exposure

IPEC-J2 cells were grown in complete culture medium (Dulbecco’s modified Eagle’s medium: F12 medium = 1:1, 10% fetal calf serum; Gibco BRL, Life Technologies, New York, NY, USA), and seeded into 12-well plates at 5.0 × 10^5^ cells per well, prior to a 24 h incubation in a humid chamber at 37 °C and 5% CO_2_. *E. coli* F18ab {107/86 (O139:K12:H1)} and *E. coli* F18ac {2134P (O157:H19)} fimbriae standard strains were obtained from the Institute of Microbiology, University of Pennsylvania. *E. coli* F18 strains were introduced to the LB culture medium, and maintained on a rocking platform for 12 h at 200 r/min. Next, 1 × 10^9^ CFU of *E. coli* bacteria were introduced to a monolayer of about 5 × 10^5^ IPEC-J2 cells in each well of a 12-well culture plate (Corning, NY, USA) for 3 h at 37 °C. To obtain the two forms of *E. coli*, the cultures were centrifuged for 5 min at 4000 rpm, followed by supernatant filtration (pore size, 0.22 μm), bacterial resuspension, and three wash cycles.

### 4.5. In Vitro Adherence Assays

Adherence assays of *E. coli* F18ac-fimbriae to IPEC-J2 cells in vitro were carried out as described previously [49]. To evaluate the adhesive capacity of *E. coli*, a relative quantification technique [50], along with colony counting, scanning electron microscopy (SEM), indirect immunofluorescence and gram staining were employed as previously reported [4].

### 4.6. qPCR Analysis

Trizol (Invitrogen, Shanghai, China) was used to extract total RNAs from duodenal tissues or IPEC-J2 cells. Next, the reverse transcription of RNA was conducted using a Superscript First-Strand Synthesis system (Promega, Madison, WI, USA). SYBR Green PCR Master Mix (Applied Biosystems, Foster City, CA, USA) was employed to conduct qPCR analysis. All qRT-PCR assay primers are summarized in Table S1.

### 4.7. Western Blotting

Total protein isolation was performed with an NE-PER kit (Nuclear and Cytoplasmic Extraction Reagents) (Thermo Fisher Scientific, New York, NY, USA). A bicinchoninic acid (BCA, Nanjing Keygen Biotech, Nanjing, China) kit was employed to normalize protein levels. Proteins were transferred to PVDF membranes and immunoblotted with antibodies against WTAP, GCNT2 and YTHDF2. Horseradish peroxidase-labeled antibody (HRP, 1:5000) was employed as secondary antibody, and GAPDH and HSP90 protein were the endogenous controls.

### 4.8. Indirect Immunofluorescence (IFA)

IPEC-J2 cells (*E. coli* F18 exposure vs. control) were carefully rinsed with PBS, prior to fixation in 4% paraformaldehyde, and treatment with Triton X-100. Subsequently, the cells were treated with goat-blocking serum for 1 h, followed by incubation with WTAP antibody (1:500), and secondary antibody IgG-HRP (1:3000). Next, they were treated with DAPI (1:1000), and thrice rinsed with PBS before mounting on slides with a fade-resistant fluorescent mounting medium, prior to analysis via confocal microscopy.

### 4.9. Transcriptome Sequencing

To conduct transcript sequencing, the total RNA was extracted from normal (Control, *n* = 3), *E. coli* F18-exposed (*E. coli*, *n* = 3), negative control (shCtrl, *n* = 3), and *WTAP* knockdown (shWTAP, *n* = 3) IPEC-J2 cells. All RNA samples were converted to double-stranded cDNA, which were then sequenced on the Illumina Hiseq2500 platform by Oebiotech Corporation (Shanghai, China). The transcript sequencing data were then uploaded to the NCBI SRA repository under the BioProject IDs: PRJNA826808, PRJNA826988.

### 4.10. RNA Knockdown and Overexpression

In case of *WTAP*, *GCNT2, YTHDF2*, small interfering RNA (siRNA) vector and negative control (siCtrl) (Table S2) were chemically synthesized by Ribobio (Guangzhou, China), and incorporated into cultured IPEC-J2 cells, along with Lipofectamine 2000 from Invitrogen (Waltham, MA, USA). Overexpressing vector (pGLV5-YTHDF2) and a negative control (pGLV5-NC) were synthesized by GenePharma (Shanghai, China), and incorporated into cultured IPEC-J2 cells.

### 4.11. Methylated RNA Immunoprecipitation (MeRIP)-qPCR

MeRIP assay was employed based on a previously published protocol [51]. In brief, following total RNAs extraction from IPEC-J2 cells, the fragmented RNAs were treated with magnetic Dynabeads associated with anti-m^6^A antibody to detect transcripts with m^6^A. The beads were then treated with Proteinase K, and RNA was isolated for verification via qPCR. The primer sequences that amplified the m^6^A peak region are the following: GCNT2-m^6^A-3294, F: CAAGCCTGTGTTGACTGTTTCTTGT; R: TCACTCTGATTTAGGGTTCTTTCTC. GCNT2-m^6^A-3467, F: TGTGAAATGTTTGTCTGGCACA; R: AGAATGCTTCCTCTAGCACTGT

### 4.12. Dual-luciferase Reporter (DLR) Assays

Wild-type (WT) or mutant-type (Mut) *GCNT2*-3′UTR (chr7: 7419012-7419243) were ligated downstream of the pmirGLO Dual-Luciferase vector. To conduct DLR assay, cells were seeded in 24-well plates and co-incorporated with wild-type (GCNT2-WT) or mutant-type (GCNT2-Mut) and siCtrl (or siWTAP, or siYTHDF2). Then, the firefly and Renilla bioluminescence were recorded using DLR Gene Assay Kit (Beyotime, Shanghai, China). Data normalization was performed via computation of the ratio between firefly and Renilla luciferase activities.

*GCNT2*-3’UTR with WT m^6^A sites:

CGGCATCTGTATCTATGGAAACGGAGACTTAAAGTGGCTGATGAATTCATCAAGCCTCTTTGCTAACAAGTTTGAGCTCAGTACCTACCCTCTTACCGTGGAATGCCTAGA**A**CTGAGGCTTCGAGAAAGAACCCTAAATCAGAGTGAAATTGAAATACAGCCCAGCTGGTATTTTTGATTGGCTGCCACTCACAGGTGAAGGGAAATCACAGCTGGGAAGGAAAACCTTTCT

*GCNT2*-3’UTR with Mut m^6^A sites:

CGGCATCTGTATCTATGGAAACGGAGACTTAAAGTGGCTGATGAATTCATCAAGCCTCTTTGCTAACAAGTTTGAGCTCAGTACCTACCCTCTTACCGTGGAATGCCTAGA**T**CTGAGGCTTCGAGAAAGAACCCTAAATCAGAGTGAAATTGAAATACAGCCCAGCTGGTATTTTTGATTGGCTGCCACTCACAGGTGAAGGGAAATCACAGCTGGGAAGGAAAACCTTTCT

### 4.13. RNA Immunoprecipitation (RIP)

The RIP assay was performed in IPEC-J2 cells according to the RNA Immunoprecipitation Kit (24T, Geneseed Biotech Co., Ltd., Guangzhou, China) following the manufacturer’s instructions. YTHDF2 antibody or rabbit IgG (as control) were used for the RIP assay. Both input and co-RIPs were extracted via Trizol (Thermo Fisher Scientific, New York, NY, USA) and assessed via qPCR analysis.

### 4.14. mRNA Stability Analysis

To assess *GCNT2* mRNA stability, the OE-Ctrl and OE-YTHDF2 IPEC-J2 cells were exposed to 10 μg/mL actinomycin D (MedChemExpress, Shanghai, China) for 0, 3 h and 6 h. Following cell harvest, total RNA was isolated for reverse transcription and qPCR analysis of *GCNT2* transcript.

### 4.15. Statistical Analysis

Data analyses were performed via Student’s t test (two-tailed) using the SPSS v.20 (IBM Corp, Armonk, NY, USA) and GraphPad Prism 6.0 software (GraphPad Inc., La Jolla, CA, USA), and are presented as mean ± SD. * *p* < 0.05 and ** *p* < 0.01 were set as the significance thresholds.

## 5. Conclusions

In conclusion, we identified the m^6^A methyltransferase WTAP as a critical regulator of *E. coli* F18 susceptibility in IPEC-J2 cells. This is a novel study that suggests that m^6^A regulates *E. coli* F18 invasion by targeting the GCNT2/glycosphingolipid biosynthesis in a YTHDF2-dependent fashion. Our findings offer a novel insight into the underlying mechanism of m^6^A modification and its regulators in IPEC-J2 cell biology. However, the in vivo data should be extended to strengthen our conclusion, such as the WTAP knockout mice models. A more comprehensive understanding of RNA modifications can enable us to establish small-molecule inhibitors or gene therapy approaches that target proteins to control gene expression or protein translation. Our research will provide theoretical guidance that will help resolve the challenge of combating bacterial diarrhea in piglets.

## Data Availability

The data presented in this study are available on request from the corresponding author.

## Supplementary Materials

The following supporting information can be downloaded at: https://www.mdpi.com/article/10.3390/ijms232113602/s1.

## Abbreviations

m^6^A	N^6^-methyladenosine
PWD	post-weaning diarrhea
*E. coli*	*Escherichia coli*
IPEC-J2	intestinal porcine epithelial cell
IFA	indirect immunofluorescence assay
MeRIP	methylated RNA immunoprecipitation
SEM	scanning electron microscopy
UTR	untranslated regions
DGEs	differential expression genes
YTHDF	N^6^-methyladenosine RNA binding protein 2
WTAP	Wilms’ tumor 1-associating protein
GCNT2	N-acetyllactosaminide beta-1,6-N-acetylglucosaminyl-transferase
