# Supplementary material for: Comprehensive Analysis Revealed the Potential Roles of N6-Methyladenosine (m6A) Mediating E. coli F18 Susceptibility in IPEC-J2 Cells
Source: Int J Mol Sci. 2022 Nov 6;23(21):13602. doi: 10.3390/ijms232113602 (PMC9654476; doi:10.3390/ijms232113602)
Supplement: Supplementary file 1 [file ijms-23-13602-s001.zip › ijms-1922138-supplementary.pdf]

Supporting information

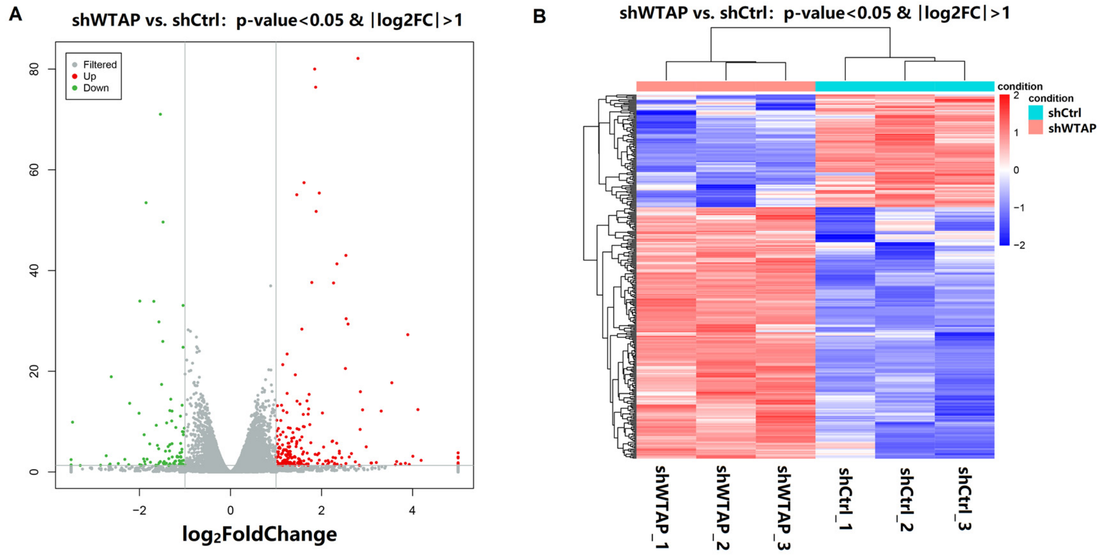

Figure S1. RNA-seq analysis in IPEC-J2 cells after WTAP knockdown. (A) A volcano plot displaying the differentially expressed genes (DEGs) between the WTAP knockdown (shWTAP) and control group (shCtrl). **(B)** Hierarchical clustering analysis of DEGs from shWTAP and shCtrl cells.

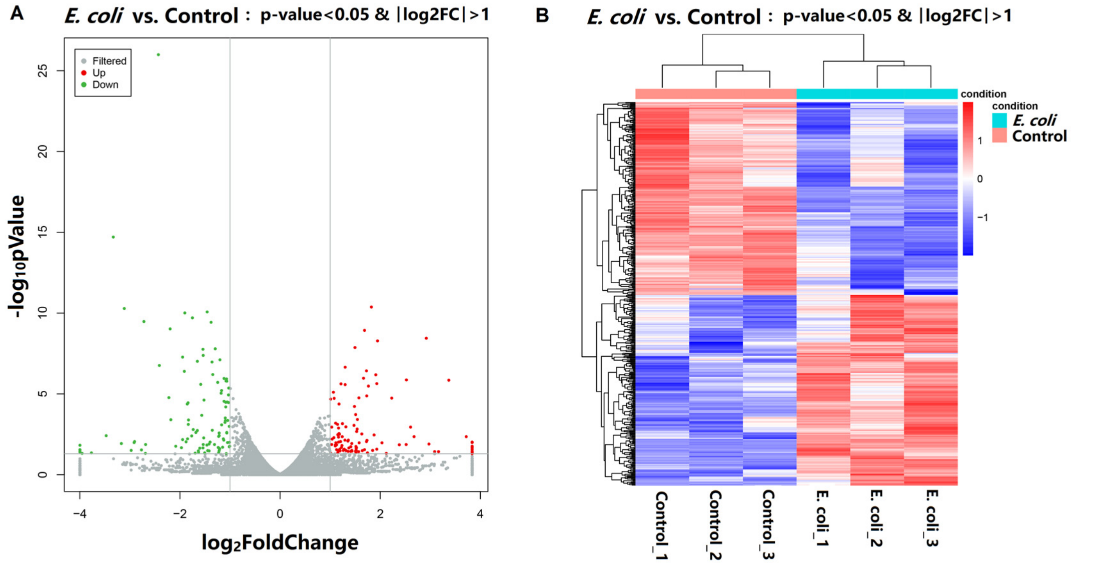

Figure S2. RNA-seq analysis in IPEC-J2 cells after *E. coli* F18 infection. (A) A volcano plot displaying the differentially expressed genes (DEGs) between the *E. coli* F18 infection (*E. coli*) and normal group (Control). **(B)** Hierarchical clustering analysis of DEGs from *E. coli* and Control cells.

Table S1. Real-time PCR primers and sequences

Gene	Accession number	Primer（5’→3’）	Length（bp）
METTL3	XM_003128580.5	F: CCCTATGGGACCCTGACAGA<br>R: TGACACCAACCAAGCAGTGT	250
WTAP	NM_001244241.1	F: TCCATTCGTCTTTCCTCTCCG<br>R: GCCTCACTCAGTCGAACCTTT	130
FTO	NM_001112692.1	F: GATCTCAATGCCACCCACCA<br>R: CCACTCAAACTCGACCTCGT	237
METTL14	XM_003129231.6	F: GGGAGAGTGTGTTTACGCAAG<br>R: TGAAGTCCCCGTCTGTGCTA	184
ALKBH5	XM_021067995.1	F: CCCCATCCACATCTTCGAGC<br>R: CGCATCTAACCTTGTCTTCCTGA	250
GCNT2	XM_021100168.1	F: ACGCTTAATAGGATTCCAGGTGT<br>R: CCCACCAGGTAGTTCACCATT	133
YTHDF2	XM_005665152.3	F: ATGGTAACAAGAGACTGGATGCTG<br>R: ATGTGTCGCAGTTGGCTATTGG	229
PILIN	M25302.1	F: AGGCCGAACCAAAGAAGCAT<br>R: TCACCATCAGGGTTTCTGAGT<br>R: CGTGGCTAGTTCAAGTGGTAAG	117
ABO	NM_213799.1	F: TACCCGAAGTACCCAGGTTGA<br>R: CCTGTGTCCCACCATGAAGT	245
FUT2	U70881.2	F: AATCCCTGACCTCACTCCGTG<br>R: CGGAACTACAACTGCTGGCC	123
FUT2A	XM_013998595.2	F: TCCATCATCTTCCACTGCCG<br>R: CGTTGATGGTGAACATGCCC	118
PILIN	M25302.1	F: AGGCCGAACCAAAGAAGCAT<br>R: TCACCATCAGGGTTTCTGAGT	117
β-actin	NC_010445.3	F: GTCGTACTCCTGCTTGCTGAT<br>R: CCTTCTCCTTCCAGATCATCGC	119
GAPDH	AF017079.1	F: ACATCATCCCTGCTTCTACTGG<br>R: CTCGGACGCCTGCTTCAC	188

`

Table S2. Sequence information of siRNA vector

Name	Sequence (5'→3')
si*WATP*-1	GCAAGUACACAGAUCUUAATT<br>UUAAGAUCUGUGUACUUGCTT
si*WTAP*-2	GCAAGAGUGUACUACUCAATT<br>UUGAGUAGUACACUCUUGCTT
si*WTAP*-3	GCGGGAAUAAGGCCUCCAATT<br>UUGGAGGCCUUAUUCCCGCTT
si*YTHDF2*-1	ACGUCAAGGUCGUGGGAAATT<br>UUUCCCACGACCUUGACGUTT
si*YTHDF2*-1	UGGAGAACAACGAGAAUAATT<br>UUAUUCUCGUUGUUCUCCATT
si*GCNT2*-1	GGACUUUGACACUUUCGAATT<br>UUCGAAAGUGUCAAAGUCCTT
si*GCNT2*-2	GCUCUUCAGGGCUGUCUAUTT<br>AUAGACAGCCCUGAAGAGCTT
si*GCNT2*-3	GCUAUUAAGCGGACGAAAUTT<br>AUUUCGUCCGCUUAAUAGCTT
siCtrl	UUCUCCGAAACGUGUCACGUTT<br>ACGUGACACGUUCGGAGAATT

Table S3. Common significant pathways of differentially expressed genes (DEGs) from shWTAP vs. shCtrl and *E. coli* vs. Control (*P*-value<0.05)

Pathway ID	Name	DEGs
ko04610	Complement and coagulation cascades	*C5; C1S; CFB; THBD; F3; PLAU; C3; SERPINB2; PLAUR; C2; F2; C4BPA; CR1*
ko04976	Bile secretion	*CYP7A1; SLC51A; SLC10A2; AQP1; ABCC2; SULT2A1*
ko04145	Phagosome	*FCGR1A; SLA-DRB1; SLA-DRA; SLA-DQA1; C3; CTSL; RAB7B; ITGAV; THBS1; ATP6V0D2*
ko04514	Cell adhesion molecules (CAMs)	*SLA-DRB1; SLA-DRA; SLA-DQA1; CLDN8; CLDN9; NTNG2; ITGAV; VCAM1; SPN; VTCN1*
ko00601	Glycosphingolipid biosynthesis -lacto and neolacto series	*GCNT2*
ko00140	Steroid hormone biosynthesis	*HSD3B1; CYP7A1; HSD17B2; HSD11B1*
ko00950	Isoquinoline alkaloid biosynthesis	*LOC100525099; DDC*
ko04978	Mineral absorption	*TF; TRPM7; SLC5A1*
ko00380	Tryptophan metabolism	*LOC100525099; IDO2; AOX2; DDC*
ko00360	Phenylalanine metabolism	*LOC100525099; DDC*
ko04974	Protein digestion and absorption	*KCNN4; SLC7A8; COL15A1; COL5A1; COL3A1; COL12A1*
